# Optimal target blood pressure in elderly with septic shock (OPTPRESS) trial: study protocol for a randomized controlled trial

**DOI:** 10.1186/s13063-022-06732-9

**Published:** 2022-09-24

**Authors:** Akira Endo, Kazuma Yamakawa, Takashi Tagami, Yutaka Umemura, Kyosuke Takahashi, Hiroki Nagasawa, Yuichi Araki, Mitsuaki Kojima, Toshiki Sera, Masayuki Yagi, Ryo Yamamoto, Jiro Takahashi, Masaki Nakane, Chikashi Takeda, Chihiro Narita, Satoshi Kazuma, Hiroko Okura, Hiroyuki Takahashi, Takeshi Wada, Shu Tahara, Ayaka Matsuoka, Todani Masaki, Atsushi Shiraishi, Keiichiro Shimoyama, Yuta Yokokawa, Rintaro Nakamura, Hisako Sageshima, Yuichiro Yanagida, Kunihiko Takahashi, Yasuhiro Otomo

**Affiliations:** 1 Trauma and Acute Critical Care Center, Tokyo Medical and Dental University Hospital, 1-5-45 Yushima, Bunkyo-ku, Tokyo, Japan; 2 Department of Acute Critical Care Medicine, Tsuchiura Kyodo General Hospital, 4-1-1 Otsuno, Tsuchiura, Ibaraki, Japan; 3 Department of Emergency Medicine, Osaka Medical and Pharmaceutical University, 2-7 Daigakumachi, Takatsuki, Osaka, Japan; 4 Department of Emergency and Critical Care Medicine, Nippon Medical School Musashikosugi Hospital, 1-396 Kosugimachi, Nakahara-ku, Kawasaki, Kanagawa Japan; 5 Division of Trauma and Surgical Critical Care, Osaka General Medical Center, 3-1-56 Bandai-Higashi, Sumiyoshi, Osaka, 558-8558 Japan; 6 Emergency and Critical Care Center, Hyogo Prefectural Nishinomiya Hospital, 13-9, Rokutanjicho, Nishinomiya City, Hyogo Japan; 7 Department of Acute Critical Care Medicine, Shizuoka Hospital, Juntendo University, 1129 Nagaoka, Izunokuni City, Shizuoka, Japan; 8 Emergency and Critical Care Medicine, Tokyo Women’s Medical University Adachi Medical Center, 4-33-1 Kohoku, Adachi-ku, Tokyo, Japan; 9 Critical Care Medical Center, Hiroshima Prefectural Hospital, 1-5-54 Ujinakanda, Minami-ku, Hiroshima, Japan; 10 Emergency Medicine and Acute Care Surgery, Matsudo City General Hospital, 993-1 Sendabori, Matsudo-shi, Chiba, Japan; 11 Department of Emergency and Critical Care Medicine, Keio University School of Medicine, 35 Shinanomachi, Shinjuku, Tokyo, Japan; 12 Department of Acute Medicine, Kawasaki Medical School, 577 Matsushima, Kurashiki, Okayama, Japan; 13 Department of Emergency and Critical Care Medicine, Yamagata University Hospital, 2-2-2 Iida-nishi, Yamagata, Japan; 14 Department of Anesthesia, Kyoto University Hospital, Shogoin Kawaharacho54, Sakyo-ku, Kyoto, Japan; 15 Department of Emergency Medicine, Shizuoka General Hospital, 4-27-1 Kitaando, Aoiku, Shizuoka, Japan; 16 Department of Intensive Care Medicine, Sapporo Medical University School of Medicine, South 1, West 16, Chuo-ku, Sapporo, Hokkaido Japan; 17 Department of Emergency and Critical Care Medicine, Faculty of Medicine, Fukuoka University, 7-45-1 Nanakuma, Jonan-ku, Fukuoka, Japan; 18 Emergency and Critical Care Medical Center, Teine Keijinkai Hospital, 1-jo 12-chome, Maeda, Teine-ku, Sapporo, Japan; 19 Department of Anesthesiology and Critical Care Medicine, Division of Acute and Critical Care Medicine, Hokkaido University Faculty of Medicine, N15, W7, Kita-ku, Sapporo, Japan; 20 Department of Emergency Medicine, Sunagawa City Medical Center, 3-chome1-1 Nishi 4-jo Kita, Sunagawa-shi, Hokkaido Japan; 21 Department of Emergency and Critical Care Medicine, Faculty of Medicine, Saga University, 5-1-1 Nabeshima, Saga City, Saga, Japan; 22 Advanced Medical Emergency and Critical Care Center, Yamaguchi University Hospital, 1-1-1 Minami-Kogushi, Ube, Yamaguchi, Japan; 23 Emergency and Trauma Center, Kameda Medical Center, 929 Higashicho, Kamogawa, Chiba, Japan; 24 Department of Emergency and Critical Care Medicine, Tokyo Medical University, 6-7-1, Nisi-Shinjuku, Shinjuku-Ku, Tokyo, 160-0023 Japan; 25 Division of Emergency and Critical Care Medicine, Tohoku University Graduate School of Medicine, 1-1 Seiryocho, Aoba-ku, Sendai, Miyagi Japan; 26 Department of Emergency Medicine, Ibaraki Seinan Medical Center Hospital, 2190 Sakai, Sashima, Ibaraki, Japan; 27 Department of Emergency Medicine, Sapporo City General Hospital, 13-1-1, Kita-11-jonishi, Chuo-ku, Hokkaido Japan; 28 Department of Emergency, Fukuoka City Hospital, 13-1 Yoshitukahoncho, Hakata-ku, Fukuoka, Japan; 29 M&D Data Science Center, Tokyo Medical and Dental University, 1-5-45 Yushima, Bunkyo-ku, Tokyo, Japan

**Keywords:** Arterial pressure, Geriatrics, Hypotension, Sepsis, Vasoconstrictor agents

## Abstract

**Background:**

Hemodynamic stabilization is a core component in the resuscitation of septic shock. However, the optimal target blood pressure remains debatable. Previous randomized controlled trials suggested that uniformly adopting a target mean arterial pressure (MAP) higher than 65 mmHg for all adult septic shock patients would not be beneficial; however, it has also been proposed that higher target MAP may be beneficial for elderly patients, especially those with arteriosclerosis.

**Methods:**

A multicenter, pragmatic single-blind randomized controlled trial will be conducted to compare target MAP of 80–85 mmHg (high-target) and 65–70 mmHg (control) in the resuscitation of septic shock patients admitted to 28 hospitals in Japan. Patients with septic shock aged ≥65 years are randomly assigned to the high-target or control groups. The target MAP shall be maintained for 72 h after randomization or until vasopressors are no longer needed to improve patients’ condition. To minimize the adverse effects related to catecholamines, if norepinephrine dose of ≥ 0.1 μg/kg/min is needed to maintain the target MAP, vasopressin will be initiated. Other therapeutic approaches, including fluid administration, hydrocortisone use, and antibiotic choice, will be determined by the physician in charge based on the latest clinical guidelines. The primary outcome is all-cause mortality at 90 days after randomization.

**Discussion:**

The result of this trial will provide great insight on the resuscitation strategy for septic shock in the era of global aged society. Also, it will provide the better understanding on the importance of individualized treatment strategy in hemodynamic management in critically ill patients.

**Trial registration:**

UMIN Clinical Trials Registry; UMIN000041775. Registered 13 September 2020.

**Supplementary Information:**

The online version contains supplementary material available at 10.1186/s13063-022-06732-9.

## Administrative information

**Table:** 

Title {1}	Optimal Target Blood Pressure in Elderly with Septic Shock (OPTPRESS) trial: study protocol for a Randomized Controlled Trial
Trial registration {2a and 2b}.	UMIN Clinical Trials Registry; UMIN000041775. Registered 13 September 2020.
Protocol version {3}	The latest protocol is ver.3 that has been approved on 17 September 2021
Funding {4}	This work was supported by the Japan Society for the Promotion of Science KAKENHI (grant number: JP21H03197), which had no role in the design of the study and collection, analysis, and interpretation of data and in writing the manuscript
Author details {5a}	^1^Trauma and Acute Critical Care Center, Tokyo Medical and Dental University Hospital, 1–5-45 Yushima, Bunkyo-ku, Tokyo, Japan ^2^Department of Acute Critical Care Medicine, Tsuchiura Kyodo General Hospital, 4–1-1 Otsuno, Tsuchiura, Ibaraki, Japan ^3^Department of Emergency Medicine, Osaka Medical and Pharmaceutical University, 2–7 Daigakumachi, Takatsuki, Osaka, Japan ^4^Department of Emergency and Critical Care Medicine, Nippon Medical School Musashikosugi Hospital, 1–396 Kosugimachi, Nakahara-ku, Kawasaki, Kanagawa, Japan ^5^Division of Trauma and Surgical Critical Care, Osaka General Medical Center, 3–1-56 Bandai-Higashi, Sumiyoshi, Osaka 558–8558, Japan ^6^Emergency and Critical Care Center, Hyogo Prefectural Nishinomiya Hospital, 13–9, Rokutanjicho, Nishinomiya City, Hyogo, Japan ^7^Department of Acute Critical Care Medicine, Shizuoka Hospital, Juntendo University, 1129 Nagaoka, Izunokuni City, Shizuoka, Japan ^8^Emergency and Critical Care Medicine, Tokyo Women’s Medical University Adachi Medical Center, 4–33–1 Kohoku, Adachi-ku, Tokyo, Japan ^9^ Critical Care Medical Center, Hiroshima Prefectural Hospital, 1–5-54 Ujinakanda, Minami-ku, Hiroshima, Japan ^10^Emergency Medicine and Acute Care Surgery, Matsudo City General Hospital, 993–1 Sendabori, Matsudo-shi, Chiba, Japan ^11^Department of Emergency and Critical Care Medicine, Keio University School of Medicine, 35 Shinanomachi, Shinjuku, Tokyo, Japan. ^12^Department of Acute Medicine, Kawasaki Medical School, 577 Matsushima, Kurashiki, Okayama, Japan. ^13^Department of Emergency and Critical Care Medicine, Yamagata University Hospital, 2–2-2 Iida-nishi, Yamagata, Japan ^14^Department of Anesthesia, Kyoto University Hospital, Shogoin Kawaharacho54, Sakyo-ku, Kyoto, Japan ^15^Department of Emergency Medicine, Shizuoka General Hospital, 4–27–1 Kitaando, Aoiku, Shizuoka, Japan ^16^ Department of Intensive Care Medicine, Sapporo Medical University School of Medicine, South 1, West 16, Chuo-ku, Sapporo, Hokkaido, Japan ^17^Department of Emergency and Critical Care Medicine, Faculty of Medicine, Fukuoka University, 7–45–1 Nanakuma, Jonan-ku, Fukuoka, Japan ^18^Emergency and Critical Care Medical Center, Teine Keijinkai Hospital, 1-jo 12-chome, Maeda, Teine-ku, Sapporo, Japan. ^19^Division of Acute and Critical Care Medicine, Department of Anesthesiology and Critical Care Medicine, Hokkaido University Faculty of Medicine, N15, W7, Kita-ku, Sapporo Japan. ^20^Department of Emergency Medicine, Sunagawa City Medical Center, 3-chome1–1 Nishi 4-jo Kita, Sunagawa-shi, Hokkaido, Japan. ^21^Department of Emergency and Critical Care Medicine, Faculty of Medicine, Saga University, 5–1-1 Nabeshima, Saga City, Saga, Japan ^22^Advanced Medical Emergency and Critical Care Center, Yamaguchi University Hospital, 1–1-1 Minami-Kogushi, Ube, Yamaguchi, Japan ^23^Emergency and Trauma Center, Kameda Medical Center, 929 Higashicho, Kamogawa, Chiba, Japan ^24^Department of Emergency and Critical Care Medicine, Tokyo Medical University, 6–7-1, Nisi-Shinjuku, Shinjuku-Ku, Tokyo 160–0023, Japan. ^25^Division of Emergency and Critical Care Medicine, Tohoku University Graduate School of Medicine, 1–1 Seiryocho, Aoba-ku, Sendai, Miyagi, Japan ^26^Department of Emergency Medicine, Ibaraki Seinan Medical Center Hospital, 2190 Sakai, Sashima, Ibaraki, Japan ^27^Department of Emergency Medicine, Sapporo City General Hospital, 13–1-1, Kita-11-jonishi, Chuo-ku, Hokkaido, Japan. ^28^Department of Emergency, Fukuoka City Hospital, 13–1 Yoshitukahoncho, Hakata-ku, Fukuoka, Japan ^29^M&D Data Science Center, Tokyo Medical and Dental University, 1–5-45 Yushima, Bunkyo-ku, Tokyo, Japan
Name and contact information for the trial sponsor {5b}	This trial is an investigator-initiated clinical trial funded by the Japan Society for the Promotion of Science (JSPS). https://www.jsps.go.jp/english/index.html Kojimachi Business Center Building, 5–3-1 Kojimachi, Chiyoda-ku, Tokyo 102–0083 https://reg34.smp.ne.jp/regist/is? SMPFORM = minf-oaojo-6037a4581743cdea076b4466da3c128e
Role of sponsor {5c}	The JSPS does not influence the study design; collection, management, analysis, and interpretation of data; writing of the report; and the decision to submit the report for publication.

## Introduction

### Background and rationale {6a}

Sepsis is a leading cause of death worldwide. The mortality rate of septic shock is reported to be approximately 52.1% when diagnosed using the Sepsis-3 criteria [1]. Initial resuscitation targeting hemodynamic stabilization to optimize regional tissue perfusion of vital organs and maintain oxygen metabolism is a core component of treatment in patients with septic shock. The Surviving Sepsis Campaign (SSC) guidelines 2021 recommends an initial target mean arterial pressure (MAP) of 65 mmHg for adults with septic shock on vasopressors [2]. However, this target MAP has not been supported by sufficient evidence; the optimal target blood pressure is still under debate [3–6].

Previous randomized controlled trials did not show any survival benefits when target MAP was higher than 65–70 mmHg, whereas there was a reduction in the frequency of renal replacement therapy among patients with known hypertension [7, 8]. These results suggest that uniformly adopting a higher target MAP for all adult patients with septic shock, including young individuals with no comorbidities, would not be beneficial. Catecholamine-related adverse effects might have affected the outcome and outweighed the therapeutic effects of maintaining higher blood pressure [9, 10]. In addition, a recent trial comparing vasodilatory shock patients aged 65 years or older found a tendency for lower mortality in the group targeting MAP of 60–65 mmHg [11]; however, the proportion of septic shock patients was less than half. Since the tissue oxygen demand in septic shock is elevated due to excessive metabolic status and mitochondrial dysfunction, stricter hemodynamic management might be required in patients with septic shock [12, 13].

It has also been proposed that a higher target MAP may be beneficial for elderly patients, particularly those with arteriosclerosis [3, 4]. Individualized management of intraoperative blood pressure for high-risk patients resulted in a significant reduction in postoperative organ dysfunction compared to the standard management, [14] suggesting that optimal target blood pressure for patients requiring intensive respiratory and circulatory management may differ depending on the patient’s background [15].

### Objectives {7}

In light of these findings, there is a need to examine the effectiveness of higher blood pressure management for septic shock patients with chronic hypertension using strategies that minimize the catecholamine dose. We hypothesized that elderly patients with septic shock, who generally have been shown to have high blood pressure under normal conditions, would benefit from management using a higher blood pressure target.

### Trial design {8}

The trial design is a multicenter, pragmatic single-blind randomized controlled trial.

## Methods: participants, interventions, and outcomes

### Study setting {9}

The ‘optimal target blood pressure in elderly with septic shock (OPTPRESS)’ trial is a multicenter, pragmatic single-blind randomized controlled trial which will be conducted in patients with septic shock aged 65 years or older. In this trial, the subjects are randomly assigned to either of the following two groups: (i) target MAP = 80–85 mmHg (high-target group) or (ii) target MAP = 65–70 mmHg (control group). Although it is ideal to include only patients with chronic hypertension, in actual clinical settings, information on normal blood pressure is unavailable for many patients who are brought to the emergency room for fatal conditions. Therefore, to make the trial design more practical in the real world, potential chronic hypertensive patients are included based on their age. The cut-off value of age was determined based on population-based surveys from Japan and other countries [16, 17]. These surveys included individuals with or without documented chronic hypertension regardless of antihypertensive medication and showed that the MAP of individuals aged more than 65 years is generally around 90 mmHg in normal conditions. Twenty-eight hospitals in Japan are participating in the OPTPRESS trial, in which universities, non-universities, urban, rural, public, and private hospitals coexist (Table S1 in the Additional file 1).

### Eligibility criteria {10}

**Fig. 1:**
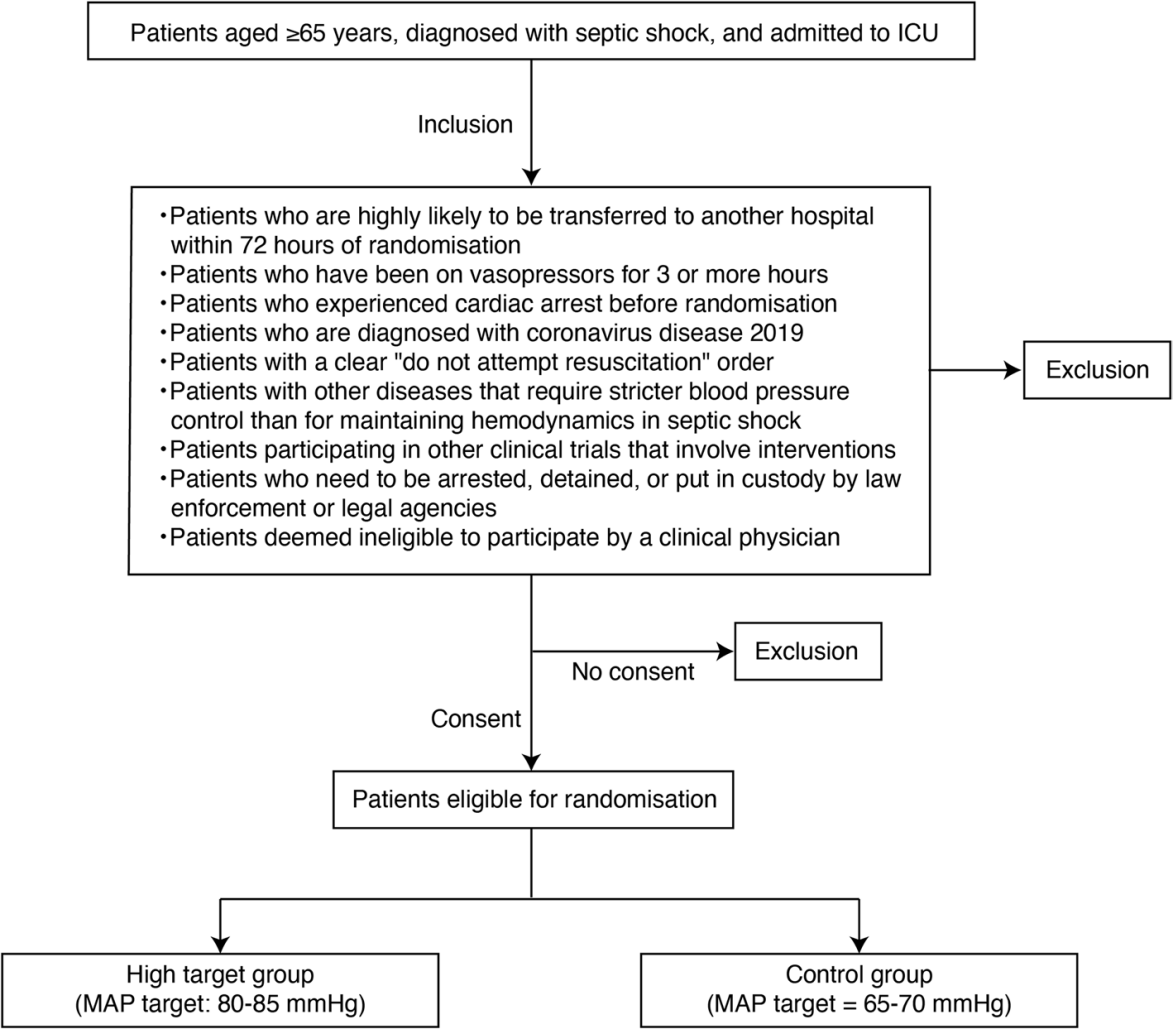
Flowchart of the patient enrolment process. ICU, intensive care unit; MAP, mean arterial pressure

A flowchart of the patient enrolment process is presented in Fig. 1. Individuals who meet all the following criteria will be included:

(i) Patients who are aged 65 years or older
(ii) Patients who are diagnosed with septic shock clinically
(iii) Patients who admitted to intensive care unit

The place of onset of septic shock (i.e., inside or outside the hospital) does not matter. Sepsis and septic shock are defined according to the Sepsis-3 standard, [18] in which septic shock is defined as persistent hypotension requiring vasopressors to maintain an MAP ≥ 65 mmHg and a serum lactate level greater than 2 mmol/L despite adequate fluid resuscitation. Adequate initial fluid replacement according to the guidelines is “administration of at least 30 ml/kg of a crystalloid solution within 3 h of sepsis diagnosis,” but because of recent findings suggesting harmful effects from excessive fluid replacement, [19, 20] the decision of what is “adequate” is left to the clinician.

Patients who met either of the following criteria will be excluded:

(i) Patients who are highly likely to be transferred to another hospital within 72 h of randomization
(ii) Patients who have been on vasopressors for 3 or more hours
(iii) Patients experiencing cardiac arrest before randomization
(iv) Patients who are diagnosed with coronavirus disease 2019
(v) Patients who have the advanced directives restricting implementation of the standard critical care (e.g., catecholamine use, mechanical ventilation, and renal replacement therapy)
(vi) Patients with other diseases that require stricter blood pressure control than for maintaining hemodynamics in septic shock (uncontrollable active bleeding, aneurysms, arterial dissection, etc.)
(vii) Patients participating in other clinical trials that involve interventions
(viii) Patients who need to be arrested, detained, or put in custody by law enforcement or legal agencies
(ix) Patients who refuse to participate in the trial
(x) Patients deemed ineligible to participate by a clinical physician

Any patient who is ultimately unable to provide consent, either by themselves or through a legally authorized representative, will also be excluded from the analysis. The present trial is designed to randomize patients within 3 h of norepinephrine administration, as a previous meta-analysis suggested the potential risk of targeting higher MAP after 6 h of norepinephrine administration [21].

### Who will take informed consent? {26a}

The investigators will explain the study orally and in writing and provide the consent form approved by the institutional review board to the subjects or their legally authorized representative to obtain voluntary written consent. If written consent cannot be immediately obtained from a legally authorized representative, explanations and consent can be provided over the phone, and written consent will be obtained later. In case of inability to obtain consent when providing emergency resuscitation, an explanation must be provided and informed consent obtained from the patient or their legally authorized representative at a later date.

### Additional consent provisions for collection and use of participant data and biological specimens {26b}

N/A. There are no plans to collect additional participant data or biological specimens in ancillary studies.

### Interventions

#### Explanation for the choice of comparators {6b}

The subjects will be assigned to the following two groups in a 1:1 ratio: (i) high-target group (target MAP = 80–85 mmHg) or (ii) control group following SSC guidelines 2021 (target MAP = 65–70 mmHg) [2].

#### Intervention description {11a}

The target MAP in the present trial shall be maintained for 72 h after randomization or until vasopressors are no longer needed due to improvement in patient’s condition. The target MAP after 72 h will be at the discretion of the physician in charge. If surgery is performed, we will attempt to maintain the assigned target MAP during the surgery. The assigned group will not be changed for patients in whom the target MAP cannot be achieved even by increasing the vasopressor dose or through fluid replacement. Since invasive intra-arterial blood pressure can be monitored only in resource-rich intensive care units, considering the generalizability, blood pressure will be measured noninvasively on either the left or right upper arm in principle. If non-invasive blood pressure is not monitored, it will be evaluated by direct measurement of arterial pressure (priority: radial artery > femoral artery > brachial artery > others).

#### Criteria for discontinuing or modifying allocated interventions {11b}

In the high-target group, if an adverse event that is potentially related to the administration of vasopressors [bleeding requiring transfusion, myocardial infarction, arrhythmia (ventricular tachycardia, supraventricular arrhythmia that affects hemodynamics), intestinal ischemia, peripheral limb ischemia] occurs, the target MAP will be changed to 65 mmHg.

#### Strategies to improve adherence to interventions {11c}

Trial monitoring experts, who are independent of the conduct of the trial, will monitor that the trial is being conducted in accordance with the protocol.

#### Relevant concomitant care permitted or prohibited during the trial {11d}

The amount and speed of fluid administration will be left at the discretion of the physician in charge. Norepinephrine will be the first-line vasopressor; however, to minimize the adverse effects related to catecholamines, if norepinephrine dose of ≥0.1 μg/kg/min is needed to maintain the target MAP, vasopressin will be initiated and the dose can be raised up to 0.04 U/min. This hemodynamic management protocol is designed based on the findings that the early use of vasopressin, along with catecholamines, led to requirement for lower catecholamine doses and less renal replacement therapy [22, 23]. If the target MAP still cannot be maintained, the physician in charge can add another vasopressor, increase the norepinephrine dose, or add dobutamine or hydrocortisone. In principle, the physicians are required to follow the latest SSC guidelines or Japanese clinical practice guidelines for the management of sepsis [24] and try to meet the target MAP as far as possible. The decision to reduce or discontinue vasopressors is taken by the physician in charge.

The initial choice of empiric antibacterial agents, including multidrug therapy and subsequent de-escalation, will be appropriately determined by the physician in charge. The introduction of mechanical ventilation, renal replacement therapy, and the use of thrombomodulin, antithrombin, immunoglobulin formulations, and other medications will be determined by the physician in charge. Although the type and dose of analgesics, sedatives, and muscle relaxants will also be determined by the physician in charge, in principle, the treatment should be targeted to obtain a score of − 3 to 0 on the Richmond Agitation-Sedation Scale, with assessments performed at least once a day.

#### Provisions for post-trial care {30}

If trial participate suffer from health problem related to flaws in the trial protocol within 1 year after the participation, the insurance company will provide compensation.

### Outcomes {12}

#### Primary endpoint

The primary endpoint is the all-cause mortality rate at 90 days after randomization.

#### Secondary endpoints

1. Lactate clearance at 24 h calculated by lactate levels at randomization and 24 h after
2. Incidence of arrhythmia (ventricular tachycardia, supraventricular arrhythmia that affects hemodynamics) within 72 h
3. Incidence of thromboembolism (myocardial infarction, cerebral infarction, intestinal necrosis, or irreversible ischemia of peripheral limbs) within 72 h
4. Incidence of hemorrhagic events within 72 h
5. All-cause mortality rate at 28 days
6. Mortality rate from sepsis at 28 days
7. Ventilator-free days at 28 days
8. Renal replacement therapy-free days at 28 days
9. Catecholamines-free days at 28 days
10. Mortality rate from sepsis at 90 days

Adverse events, such as arrhythmia or thromboembolism, will be clinically judged by the physician in charge based on laboratory data, images, and other findings. The ventilator, renal replacement therapy, and catecholamine-free days were defined as the number of days from day 1 to day 28 after randomization when the patient was alive and free from the support for at least 24 consecutive hours. If patients die within 28 days or are still supported after 28 days, zero will assigned.

### Participant timeline {13}

**Fig. 2:**
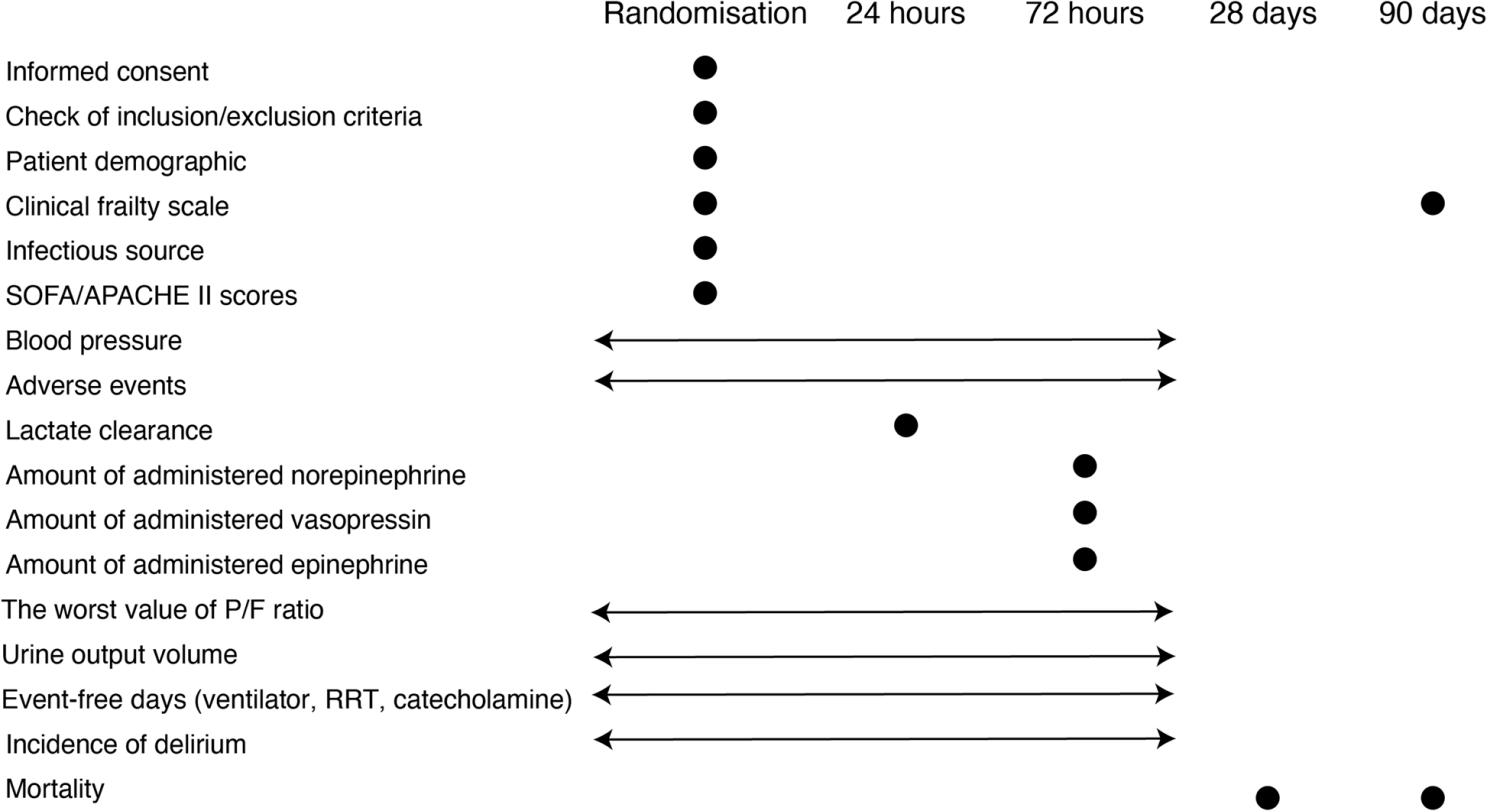
The schedule of trial assessments. SOFA, Sequential Organ Failure Assessment; APACHE, Acute Physiologic Assessment and Chronic Health Evaluation; P/F ratio, partial pressure of arterial oxygen/fraction of inspired oxygen ratio; RRT, renal replacement therapy

The schedule of the trial assessments is shown in Fig. 2.

### Sample size {14}

We hypothesized that the incidence of the primary endpoint in the control group is 45% and the absolute difference from the intervention is 10%, which was estimated based on previously reported mortality in patients with sepsis diagnosed by Sepsis-3 criteria, [1], age of included patients, and improvements in the treatment of sepsis [25]. With a 2-sided alpha level of 0.05, for a significance level and power of 0.80, each group would need 376 cases, with a total of 752 cases. Assuming that approximately 10% will drop out due to patient withdrawal or other reasons, we have calculated that approximately 836 cases will be needed.

### Recruitment {15}

To achieve the target sample size, the principal investigator will periodically disclose the cumulative number of participants per hospital to the study group and promptly convene a meeting if any problems are concerned. The principal investigator will contact the responsible person at the participating hospital, as appropriate, to monitor certainties and problems in the patient enrollment.

### Assignment of interventions: allocation

#### Sequence generation {16a}

Stratified block randomization by the presence or absence of a history of hypertension and age (< 80 years old or older) is used, and allocation will be in a 1:1 ratio to the high-target and control groups using a clinical trial data management system (HOPE eACReSS, https://acress-host2.tmd.ac.jp/gcp/index_top.htm).

#### Concealment mechanism {16b}

The mechanism of clinical trial data management system is a complete black box for all the researchers, and those who enroll patients cannot predict which group they will be assigned to. Also, the block size will not be disclosed to all the study members except for the programmer who generate the random allocation to reduce predictability of random sequence.

#### Implementation {16c}

A programmer independent of the conduct of the trial generated the allocation sequence of clinical trial data management system. A physician in a participating hospital will enroll the participants and then assign interventions via the electronic platform.

### Assignment of interventions: blinding

#### Who will be blinded {17a}

Owing to the nature of the trial, the physician in charge cannot be blinded to the patient’s assigned group. However, the analyzing statisticians will be blinded to group allocation.

#### Procedure for unblinding if needed {17b}

The analyzing statisticians will be unblinded to group allocation after all the analyses.

### Data collection and management

#### Plans for assessment and collection of outcomes {18a}

Assessment data will be recorded using an electronic trial data capture system (HOPE eACReSS, https://acress-host2.tmd.ac.jp/gcp/index_top.htm). Trial monitoring experts, who are independent of the conduct of the trial, will monitor that the data reported by the investigators is being collected accurately.

#### Plans to promote participant retention and complete follow-up {18b}

Patients will be followed-up for 90 days after randomization. If a patient is discharged from the hospital before 90 days, the investigators will contact the patients by telephone to obtain information regarding the patient’s status.

#### Data management {19}

Information on participants’ clinical data will be electronically registered through the clinical trial data management system (HOPE eACReSS, https://acress-host2.tmd.ac.jp/gcp/index_top.htm). Registered data will be regularly checked by a data monitoring committee independent of the trial and confirmed as necessary if there is a problem with the reliability of the data.

#### Confidentiality {27}

The data registered in the clinical trial data management system does not contain personal information.

#### Plans for collection, laboratory evaluation and storage of biological specimens for genetic or molecular analysis in this trial/future use {33}

N/A. There are no plans to collect or store biological specimens for genetic or molecular analysis in this trial.

### Statistical methods

#### Statistical methods for primary and secondary outcomes {20a}

The final analysis will be performed after obtaining the 90-day outcomes of the participants. In the main analysis, the intention-to-treat population is examined by comparing the difference in the 90-day mortality rates between the groups using Fisher’s exact test. The secondary analysis will include logistic regression using factors stratified at allocation as adjustment factors and survival time analysis using the time from allocation to death as the endpoint by estimating Kaplan-Meier curves, the log-rank test, adjustment analysis, and other methods. The tests will be two-tailed, and a significance level of 5% and a confidence coefficient for estimates of 95% will be considered.

For the secondary endpoints of mortality rates and incidence of adverse events, analysis of the intention-to-treat population will be the same as the primary analysis of the primary endpoint. For the secondary endpoints of lactate clearance and each organ support-free days, summary statistics will be calculated for each group in the intension-to-treat population and compared using Student’s *t*-test and other methods, as appropriate.

#### Interim analyses {21b}

The interim analysis will be conducted after 300 cases are registered when their survival/death is confirmed 90 days later. Subject registration will continue during interim analysis. In the statistical analysis, the Bayesian predictive power will be calculated for when the planned number of cases is registered based on the current data. If this declines markedly (to approximately 5% or less), we will consider discontinuing the trial. Because no decision on whether to suspend due to efficacy is made, the significance level will not be adjusted in the final analysis.

#### Methods for additional analyses (e.g. subgroup analyses) {20b}

There are no plans to conduct additional analyses. Although researchers at participating sites may conduct ancillary studies using data obtained from this trial, specific analysis methods have not been determined.

#### Methods in analysis to handle protocol non-adherence and any statistical methods to handle missing data {20c}

If there are many missing values that cannot be ignored, a sensitivity analysis will be performed using multiple imputations of the missing values.

#### Plans to give access to the full protocol, participant level-data, and statistical code {31c}

After all the ancillary analyses by the trial group, the datasets analyzed during the current study and statistical code are available from the corresponding author on reasonable request, as is the full protocol.

### Oversight and monitoring

#### Composition of the coordinating center and trial steering committee {5d}

This trial is led by the Japanese Association for Acute Medicine. The steering committee consists of four clinical researchers oversee the implementation and check the progress of the trial. A clinical research center within the principal institution designs the clinical trial data management system, including the assignment sequences, and monitor the registered data. The endpoint adjudication committee will not be established as the primary outcome is a hard outcome.

#### Composition of the data monitoring committee, its role and reporting structure {21a}

A clinical research center within the principal institution is responsible for data monitoring. They are independent of the conduct of the trial. The method will be central monitoring of data registered with electronic data capture rather than on-site monitoring. Primary endpoints, safety endpoints, deviation items, etc., will be assessed every 6 months for registered cases. Also, safety concerns, deviations, adverse event reports, etc., will be checked. The results will be notified to the principal investigator and the representative of each research participating institution. All the queries pointed out by data monitoring committee must be responded.

#### Adverse event reporting and harms {22}

Three safety monitoring experts who are independent of the conduct of the trial will monitor to ensure the reliability of the trial regarding protection of the human rights, safety, and welfare of the subjects. If the principal investigator is notified of a severe adverse event related to the trial intervention, a prompt report will be presented to the safety monitoring board and ethics review board of the principal institution. In addition, the principal investigator will take appropriate action and promptly share information on the adverse event with other investigators involved in the trial. The safety monitoring board and ethics review board of the principal institution will review and examine the report and send written recommendations made in response to the principal investigator.

#### Frequency and plans for auditing trial conduct {23}

Appropriateness of the breakdown of the research budget is reviewed for appropriateness by the Japan Society for the Promotion of Science once a year, and each expenditure is strictly checked by the audit department of the main institution that is independent of the conduct of the trial.

#### Plans for communicating important protocol amendments to relevant parties (e.g. trial participants, ethical committees) {25}

The need for protocol changes will first be discussed by the Steering Committee, followed by a meeting of representatives from each trial participating hospital. After a decision of protocol change is made, it will be discussed by the institutional review board at the principal institution. After the approval, the decision is promptly notified to relevant parties via email or other means. Any modifications will also be reflected in the trial registry.

#### Dissemination plans {31a}

After all the data collection and analyses will be complete, the findings of this trial will be presented at relevant scientific congress and disseminated through publication in a peer-reviewed journal.

## Discussion

This multicenter pragmatic randomized controlled trial named OPTPRESS trial will investigate the effectiveness of high MAP target compared to conventional MAP target in initial hemodynamic management of septic shock in potentially chronic hypertensive patients.

Since information on usual blood pressure is generally unavailable for many patients who are transferred with septic shock, to make the trial design more pragmatic, potential chronic hypertensive patients will be determined and included by their age based on the results of nationwide population-based surveys. Vasopressin is used at an early stage of shock management to minimize the adverse effects related to catecholamines. Additionally, the present trial is designed to randomize patients within 3 h of norepinephrine administration, as a previous meta-analysis suggested the potential risk of targeting higher blood pressure after 6 h of norepinephrine administration.

The major limitation of this trial is the single-blind design. Clinicians are aware of the assigned patient group, and unconscious or conscious bias is a concern. However, this does not affect the measurement of primary outcome which can be determined objectively. The accuracy of noninvasive MAP depends on the measuring method. The blood pressure in patients with shock measured by oscillometric method is said to be inaccurate. However, continuous intra-arterial blood pressure measured from invasive catheter is not always accurate due to the catheter trouble or artifact such as under- and over damping. Also, it is impractical to monitor the continuous intra-arterial blood pressure routinely especially resource-poor intensive care units in developing countries. Therefore, considering the generalizability, blood pressure will be measured noninvasively in this trial.

The result of this trial will provide novel evidence into resuscitative strategy in the management of elder septic shock in the era of global aged society. Also, it will provide the better understanding on the importance of individualized treatment strategy in hemodynamic management in critically ill patients.

## Trial status

The trial protocol ver.1 was approved on 7 December 2020. The latest protocol is ver.3 that has been approved on 17 September 2021 after minor revisions regarding the addition of participating hospitals and the clarification for the statements of exclusion criteria. The trial was initiated in 23 participating hospitals on 1 July 2021; subsequently, 5 more hospitals joined the trial. The trial is still in progress, and approximately 170 patients have been enrolled at the end of July 2022. The estimated primary completion date is December 2024.

## Supplementary Information

**Additional file 1: Table S1.** List of participating hospitals.

## Data Availability

After all the secondary analyses by the trial group, any data required to support the protocol can be supplied on request.

## Abbreviations

SSC: Surviving Sepsis Campaign
MAP: Mean arterial pressure
