# Supplementary material for: Optimal target blood pressure in elderly with septic shock (OPTPRESS) trial: study protocol for a randomized controlled trial
Source: Trials. 2022 Sep 24;23:799. doi: 10.1186/s13063-022-06732-9 (PMC9509562; doi:10.1186/s13063-022-06732-9)
Supplement: Supplementary file 1 — Additional file 1: Table S1. List of participating hospitals. [file 13063_2022_6732_MOESM1_ESM.docx]

| **Table S1 List of participating hospitals** | |
| --- | --- |
| Principal institution | |
|  | Trauma and Acute Critical Care Center, Tokyo Medical and Dental University Hospital |
| Other participating institutions | |
|  | Department of Emergency Medicine, Osaka Medical and Pharmaceutical University |
|  | Department of Emergency and Critical Care Medicine, Nippon Medical School Musashikosugi Hospital |
|  | Division of Trauma and Surgical Critical Care, Osaka General Medical Center |
|  | Emergency and Critical Care Center, Hyogo Prefectural Nishinomiya Hospital |
|  | Department of Acute Critical Care Medicine, Shizuoka Hospital, Juntendo University |
|  | Department of Acute Critical Care Medicine, Tsuchiura Kyodo General Hospital |
|  | Emergency and Critical Care Medicine, Tokyo Women’s Medical University Adachi Medical Center |
|  | Critical Care Medical Center, Hiroshima Prefectural Hospital |
|  | Emergency Medicine and Acute Care Surgery, Matsudo City General Hospital |
|  | Department of Emergency and Critical Care Medicine, Keio University School of Medicine |
|  | Department of Acute Medicine, Kawasaki Medical School |
|  | Department of Emergency and Critical Care Medicine, Yamagata University Hospital |
|  | Department of Anesthesia, Kyoto University Hospital |
|  | Department of Emergency Medicine, Shizuoka General Hospital |
|  | Department of Intensive Care Medicine, Sapporo Medical University School of Medicine |
|  | Department of Emergency and Critical Care Medicine, Faculty of Medicine |
|  | Emergency and Critical Care Medical Center, Teine Keijinkai Hospital |
|  | Division of Acute and Critical Care Medicine, Department of Anesthesiology and Critical Care Medicine |
|  | Department of Emergency Medicine, Sunagawa City Medical Center, |
|  | Department of Emergency and Critical Care Medicine, Faculty of Medicine, Saga University |
|  | Advanced Medical Emergency and Critical Care Center, Yamaguchi University Hospital |
|  | Emergency and Trauma Center, Kameda Medical Center |
|  | Department of Emergency and Critical Care Medicine, Tokyo Medical University |
|  | Division of Emergency and Critical Care Medicine, Tohoku University Graduate School of Medicine |
|  | Department of Emergency Medicine, Ibaraki Seinan Medical Center Hospital |
|  | Department of Emergency Medicine, Sapporo City General Hospital |
|  | Department of Emergency Fukuoka City Hospital |
